# Meeting Minimum ESPEN Energy Recommendations Is Not Enough to Maintain Muscle Mass in Head and Neck Cancer Patients

**DOI:** 10.3390/nu11112743

**Published:** 2019-11-12

**Authors:** Benjamin McCurdy, Sara Nejatinamini, Brock J. Debenham, Mirey Álvarez-Camacho, Catherine Kubrak, Wendy V. Wismer, Vera C. Mazurak

**Affiliations:** 1 Department of Agricultural, Food and Nutritional Science, University of Alberta, Edmonton, AB T6G 2E1, Canada; bmccurdy@ualberta.ca (B.M.); nejatina@ualberta.ca (S.N.); wwismer@ualberta.ca (W.V.W.); 2 Department of Oncology, University of Alberta, Edmonton, AB T6G 1Z2, Canada; Brock.Debenham@albertahealthservices.ca (B.J.D.); ckubrak@ualberta.ca (C.K.); 3 Alberta Health Services, Edmonton, AB T5J 3E4, Canada; alvarezc@ualberta.ca

**Keywords:** head and neck cancer, body composition, computed tomography, cachexia, skeletal muscle loss, dietary intake

## Abstract

The relationship between dietary intake and body composition changes during cancer treatment has not been well characterized. The aim of this study was to compare dietary intake at diagnosis and end of treatment in relation to changes in muscle mass and adiposity in head and neck cancer (HNC) patients. Dietary intakes (three-day food record) and body composition using computed tomography (CT) were assessed at diagnosis (baseline) and after treatment completion (post-treatment). Skeletal muscle (SM) loss was explored as a consequence of energy and protein intake in relation to the minimum and maximum European Society of Parenteral and Enteral Nutrition (ESPEN) guidelines. Higher energy intakes (kcal/kg/day) and increases in energy intake (%) from baseline to post-treatment were correlated with attenuated muscle loss (r = 0.62, *p* < 0.01; r = 0.47, *p* = 0.04, respectively). Post-treatment protein intake demonstrated a weak positive correlation (r = 0.44, *p* = 0.05) with muscle loss, which did not persist when controlling for covariates. Meeting minimum ESPEN energy guidelines (25 kcal/kg/day) did not attenuate SM loss, whereas intakes >30 kcal/kg/day resulted in fewer participants losing muscle. Greater baseline adiposity correlated with greater SM loss (*p* < 0.001). Energy intakes of 30 kcal/kg/day may be required to protect against SM loss during treatment in HNC patients. The influence of adiposity on SM loss requires further exploration.

## 1. Introduction

People with head and neck cancer (HNC) are vulnerable to a high degree of weight loss and often present with weight loss at diagnosis and prior to the initiation of treatment. Thirty to 49% of patients have weight loss >5% prior to treatment [1]. During treatment (surgery combined with radiotherapy, with or without chemotherapy), additional weight loss occurs, and 44–88% of patients record cumulative losses of 20% or more [2]. Loss of muscle in HNC patients is associated with the early termination of chemotherapy due to toxicity [3]. Nutrition impact symptoms arising from tumor location, radiation damage, and chemotoxicity are commonly experienced by HNC patients [4]. Nutrition impact symptoms exacerbate the dietary intake of HNC patients, which ultimately reduce dietary intake and increase the risk of weight loss within this tumor group [5]. If reduction in food intake is driving this weight loss, this may be corroborated through an evaluation of changes in body composition. By the same token, it is not known what caloric intake is required to ameliorate weight and muscle loss. Questions remain as to whether those who are weight stable are stable with respect to muscle mass [1].

Cancer-derived wasting has been linked to adverse clinical outcomes including poor prognosis, dose-limiting toxicities, impaired performance and immunity, and reduced quality of life [6,7]. Loss of both muscle and adipose tissue are experienced as a result of tumor-derived and/or systemic negative energy and nitrogen balance, hypoanabolism, and hypercatabolism [8,9]. Historically, weight change has been widely used to determine the risk of malnutrition and prognosis; however, lean mass is a better predictor of poor outcomes [10]. While changes in body weight during HNC treatment have been previously documented [11], components of this weight loss remain poorly characterized in HNC patients with few studies evaluating muscle loss during the cancer trajectory [1,2,3]. It has been established that conventional nutrition care is insufficient to combat cancer wasting, but anabolic potential does exist [11,12].

Several studies have sought to determine the amount of energy that would be required to protect against weight loss in HNC, but results have been inconclusive due to the heterogeneity of patients and treatment modalities [1]. The European Society for Parenteral and Enteral Nutrition (ESPEN) has established evidence-based guidelines for dietary intake for cancer patients, including guidelines for energy, protein, and micronutrient intake [13]. The ESPEN recommends energy intakes of 25–30 kcal/kg/day and protein intakes of 1.0–1.5 g/kg/day; however, these guidelines are based on low and moderate evidence, respectively [13]. Further evidence is needed to support these guidelines to optimize nutrition care and reduce malnutrition risk in the cancer population.

In this study, we aimed to determine the associations between macronutrient intakes and changes in muscle and adipose tissue during treatment identified using longitudinal computed tomography (CT) scans. The secondary objective of this study was to explore whether meeting current ESPEN guidelines protects against loss of weight and skeletal muscle.

## 2. Materials and Methods

This cohort study is an analysis of prospective data collected from three studies reporting on clinical determinants of weight loss [5], taste and smell alterations among HNC patients [14], and micronutrient status during treatment [15]. Research procedures were approved by the Health Research Ethics Board of Alberta Cancer Committee (HREBA-CC). Informed consent was obtained from all participants. Study inclusion criteria for newly diagnosed HNC patients were: (1) aged 18 years and above, (2) treatment involving radiation therapy, with or without concurrent chemotherapy and/or surgery, and (3) maintaining oral intake during the study. Data were collected at diagnosis and prior to starting treatment (baseline) and after 6–8 weeks of treatment (post-treatment). Only patients with both dietary intake and body composition measures at baseline were included in the study (*n* = 41). Anthropometric data including weight and height was collected at baseline and follow-up. Tumor and treatment data were retrieved from patients’ health records.

### 2.1. Dietary Intake Analysis

A trained researcher instructed patients on completion of the three-day dietary records collected at two study time-points using food models. Food records were evaluated by the researcher, and clarification of any recorded items in questions were queried with the patient. No intervention occurred; treatment and dietetic support was provided according to standard of care for all patients. The Canadian Nutrient File Database Analysis of the Food Processor II Nutrient Analysis Program TM (version 9: Esha Research, Salem, Oregon, USA) was used to analyze dietary records and calculate macronutrient intakes.

### 2.2. European Society of Parenteral and Enteral Nutrition Recommendations

ESPEN recommendations for energy intake are 25–30 kcal/kg body weight (BW)/day, and 1.0–1.5 g/kg BW/day for protein [13]. For this study, the minimum ESPEN recommendations were used as reference values for energy (25 kcal BW/kg/day) and protein (1.0 g/kg BW/day) to understand whether muscle loss during chemotherapy treatment could be influenced by meeting minimum recommended intake levels of macronutrients.

### 2.3. Body Composition Assessment

Body composition was analyzed using computed tomography (CT) images taken for diagnostic purposes at baseline (prior to treatment initiation) and after completion of the treatment. The third lumbar vertebrae (L3) level was chosen as a standardized landmark, as it has been identified as being highly correlated to whole body muscle mass [16,17]. CT images were assessed using a Slice-O-Matic (Slice-O-Matic version 4.3, TomoVision, Montreal, QC, Canada) as previously described [18]. Cross-sectional areas of tissues (cm^2^) were calculated by using standard Hounsfield Unit (HU) thresholds of −29 to 150 HU for skeletal muscle (SM), −150 to −50 HU for visceral adipose tissue (VAT), and −190 to −30 HU for subcutaneous adipose tissue (SAT). Mean tissue area was subsequently normalized by height to calculate indexes (cm^2^/m^2^) for skeletal muscle (SMI), total adipose tissue (TATI), visceral adipose tissue (VATI), and subcutaneous adipose tissue (SATI). Regression equations were used to estimate whole body skeletal muscle in conventional units as follows: whole body skeletal muscle mass = 0.166 * (total skeletal muscle at L3 (cm^2^)) + 2.142; r2 = 0.855 [16].

For further analysis, percentage changes in weight, SMI, TATI, SATI, and VATI were calculated. Additionally, the timing of CT scans was unique for each individual according to their evaluation and treatment schedule. To enable comparison between individuals, percent change in muscle and adipose tissue was divided by total days between the two CT scans to calculate a daily rate of change as previously described [18]. This value was multiplied by 100 to establish an index to express change in body components as a standard unit: %∆/100 d.

### 2.4. Statistical Analysis

**Figure 1:**
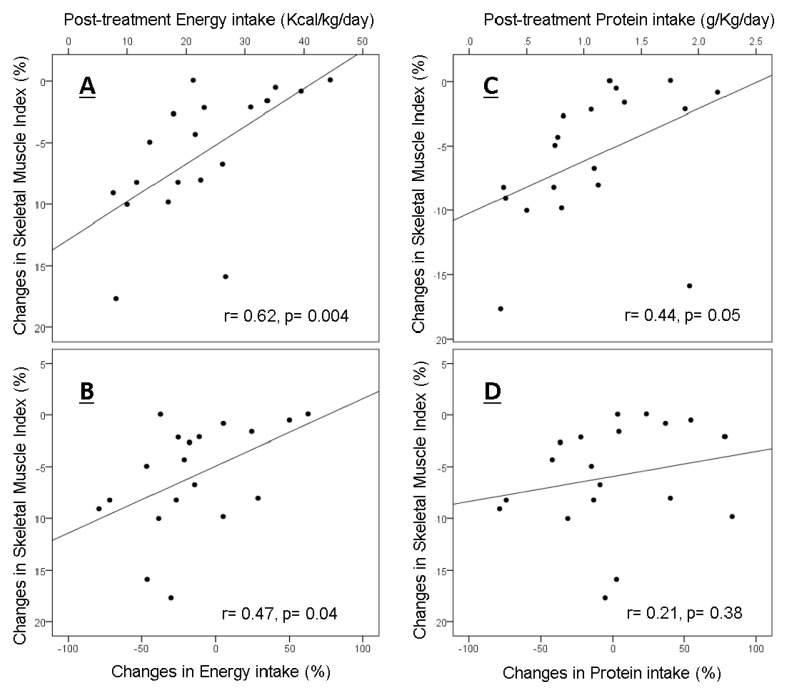
Correlations of energy and protein intakes and changes in skeletal muscle index (SMI) per 100 days. (**A**) Correlation between percent change in SMI per 100 days and post-treatment energy intake; (**B**) Correlation between percent change in SMI per 100 days and change in energy intake over treatment; (**C**) Correlation between percent change in SMI per 100 days and post-treatment protein intake; (**D**) Correlation between percent change in SMI per 100 days and change in protein intake over treatment.

Results are reported as mean ± SD, unless otherwise stated. Changes in weight, body composition depot, and nutrient intake from baseline to after treatment were analyzed using a paired sample *t*-test. The Pearson correlation coefficient was used for the association between follow-up dietary intake and change in body composition depot; then, Figure 1 was created using a simple scatterplot in SPSS. Multiple linear regression analysis was used to determine persistence of correlation significance when considering covariates. At the post-treatment time point, patients were stratified according to: (1) not meeting versus meeting or exceeding minimum ESPEN energy recommendations (25 kcal/kg BW/day); (2) not meeting versus meeting or exceeding maximum ESPEN energy recommendations (30 kcal/kg BW/day); (3) not meeting versus meeting or exceeding minimum ESPEN protein recommendations (1.0 g/kg BW/day); (4) not meeting versus meeting or exceeding maximum ESPEN protein recommendations (1.5 g/kg BW/day). Independent *t*-tests (two-tailed) were used to compare body composition depot change means between stratification groups. Statistical significance was set at *p* < 0.05 in all analyses. Statistical analyses were performed using SPSS (version 20, SPSS, Chicago, IL, USA, 2016).

## 3. Results

### 3.1. Patient Characteristics

Patient characteristics are summarized in Table 1. The majority of patients were men (*n* = 32). Mean age was 58 ± 11 years and mean body mass index (BMI) was 28.4 ± 5.1 kg/m2. The pharynx (*n* = 22; 52%) was the most common tumor site with most patients presenting with a tumor stage of three (*n* = 28; 67%). The majority of patients received chemoradiotherapy, with or without surgery (73%). Eleven patients (26%) received radiotherapy without chemotherapy (Table 1).

### 3.2. Changes in Body Composition and Dietary Intake

Body weight, SMI, TATI, SATI, and VATI significantly decreased during treatment (*p* < 0.01) (Table 2). Patients lost an average of 8.1% of pretreatment body weight (6.9 ± 4.9 kg) during treatment. Patients lost 5.6 ± 4.7%/100 d and 12.9 ± 12%/100 d of SMI and TATI during treatment, respectively. Muscle loss was estimated at −5.3 kg using an approximate equation (Shen et al., 2004). Loss of SATI and VATI were substantial during treatment (10.6 ± 11.1%/100 d and 12.3 ± 11.4%/100 d, respectively.)

The absolute amount of energy and protein intake decreased significantly over the course of treatment (*p* = 0.02). However, after normalizing dietary intake for body weight, no difference in energy or protein intake was observed between baseline and post-treatment. On average, ESPEN minimum energy and protein intake guidelines of 25 kcal/kg BW/day and 1.0 g/kg BW/day, respectively, were achieved at baseline. At the post-treatment time point, the minimum ESPEN energy intakes on average were not achieved (Table 2), with 63% of participants failing to achieve intakes satisfying ESPEN energy recommendations. Post-treatment protein intakes ranged from 0.3 g/kg BW/day to 2.2 g/kg BW/day, and 52% of participants failed to achieve intakes satisfying minimum ESPEN protein recommendations. Overall, only 14% of patients met both recommended intakes for energy and protein during the study time-points. While body weight was maintained by five patients (12%), four out of five of these patients were losing muscle. The one patient that maintained their body weight and muscle mass during the study met ESPEN guidelines for energy and protein at both time points.

### 3.3. Correlations between Post-Treatment Intakes and Changes in Skeletal Muscle

Higher intakes of energy normalized for body weight were correlated with an attenuated loss of SMI per 100 days at end of treatment (r = 0.62; *p* = 0.004) (Figure 1) and during the study (r = 0.47, *p* = 0.04), which remained significant after controlling for the covariates of age, sex, tumor stage, treatment modality, and protein intake (standardized coefficients beta = 0.65, t = 2.5, *p* = 0.025). The correlation between protein intake at the post-treatment time-point and SMI was not significant when controlling for the covariates of age, sex, tumor stage, treatment modality, and energy intake. Changes in protein intake during treatment did not correlate with SMI changes (r = 0.21; *p* = 0.38).

### 3.4. Skeletal Muscle Loss Based on Dietary Intake Stratifications

**Figure 2:**
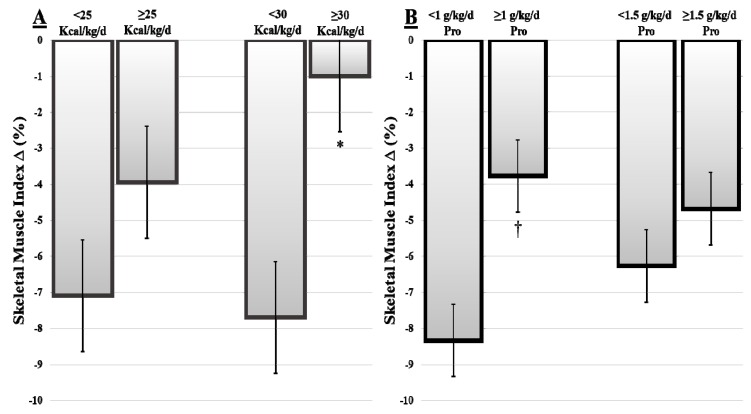
Percent changes in SMI based on post-treatment dietary intake stratifications (**A**) Percentage SM∆/100 d for 25 kcal BW/kg/day and 30 kcal/kg BW/day post-treatment energy intake stratifications; (**B**) Percentage SM∆/100 d for 1 g/kg BW/day and 1.5 g/kg BW/day post-treatment protein intake stratifications. * *p* < 0.05; † *p* = 0.05–0.06. Error bars denote standard error. Pro, protein.

No significant difference in percent muscle loss was observed when patients were categorized based on whether or not minimum recommended energy intakes were being met (<25 kcal/kg BW/day versus ≥25 kcal/kg BW/day (*p* = 0.21)) (Figure 2). However, when this stratification was increased to <30 kcal/kg BW/day and ≥30 kcal/kg BW/day, those patients with energy intakes <30 kcal/kg BW/day lost 7.6 ± 4.9%/100 d SMI, while those with intakes >30 kcal/kg BW/day lost 1 ± 0.87%/100 d SMI (*p* < 0.01). The same analysis was performed for protein. Protein intake <1.0 g/kg BW/day showed an average loss of 8.3 ± 4.3%/100 d SMI, while intakes ≥1 g/kg BW/day showed an average loss of 3.7 ± 5.0%/100 d SMI, which approached significance (*p* = 0.053). However, when the cut point of 1.5 g/kg BW/day was applied, there was no difference in percent SMI loss in those meeting or exceeding this cut point (*p* = 0.6).

### 3.5. Skeletal Muscle Loss Based on Baseline Adiposity

Patients with higher total adipose tissue at baseline lost more SMI (r = −0.75; *p* < 0.001) during treatment. Even after controlling for confounding factors (age, sex, baseline SMI, stage of disease, and type of treatment), this correlation remained significant (r = −0.78; *p* < 0.001). When VATI and SATI were evaluated separately, SATI was strongly negatively correlated (r = −0.741; *p* < 0.001), whereas VATI showed a weak negative correlation (r = −0.349; *p* = 0.054). When covariates of age, sex, tumor stage, and treatment modality were considered, those with higher VATI and SATI at baseline lost a greater amount of skeletal muscle (r = −0.57, *p* = 0.002; r = −0.78, *p* < 0.001; respectively).

## 4. Discussion

This study further confirms that weight loss and muscle wasting occur at an unchecked rate in HNC patients undergoing treatment. An average weight loss of 8% bodyweight over 6–8 weeks well exceeds the ESPEN consensus criteria for diagnosis of malnutrition [19]. By specifically quantifying muscle mass, we demonstrate that muscle loss correlates with reduced energy intake. Loss of muscle persisted when the minimum recommended intakes for energy were achieved, but were attenuated when the highest range for energy intake (30 kcal BW/kg) was met or exceeded. There was a trend toward lower SMI loss in patients who were able to meet or exceed the minimum recommended intakes for protein (1 g/kg BW/day).

Skeletal muscle index decreased by 5.7% on average during 100 days for HNC patients. Loss of this magnitude is not uncommon [10]. ESPEN indicates that muscle protein depletion, with or without adipose tissue loss, is the principle aspect of cancer-associated malnutrition [13]. As the survival rate of HNC is increasing, loss of muscle of this magnitude may be challenging to restore, particularly in patients of older age [20]. Therefore, discovering ways to combat muscle protein depletion is paramount for maintaining function and strength for survivorship.

The correlation between SMI loss and energy intake, while intuitive, was to our knowledge the first confirmation of such a relationship and suggests that energy balance plays a vital role in cancer muscle loss in HNC patients. The current consensus is that adequate protein intakes are required to overcome the anabolic resistance created by hypercatabolism and systemic inflammation experienced in the tumor-bearing state [21,22]. In the present study, energy intake was found to have a stronger correlation with SM change than that of protein intake, and a significant correlation between changes in dietary intake during treatment and SMI loss was observed only for energy intake. This confirms the requirement for adequate energy intakes to maintain muscle.

Current ESPEN guidelines recommend energy intakes and protein intakes between 25 and 30 kcal/kg BW/day and 1.0–1.5 g/kg BW/day, respectively. For HNC patients, there is a growing body of evidence to suggest that this level of intake is not adequate to prevent weight loss. In our example where two different minimum cut points were applied to our data set, the results reveal that meeting the higher level of energy is more effective at preventing weight loss. First, those not meeting compared to those meeting or exceeding the minimum recommendation reveals that SM loss was attenuated and fewer patients lost muscle mass at higher intakes. This suggests that intakes greater than 30 kcal/kg BW/day may be required to achieve muscle stability in HNC patients. Similar to energy, protein intake was first explored by those failing to meet minimum ESPEN guidelines of 1.0 g/kg BW/day compared to those meeting or exceeding the upper level of recommendations. There was a two-fold difference in amount of weight lost between patients with intakes <1.0 g/kg BW/day protein compared to patients with intakes ≥1.0 g/kg BW/day. Patients with intakes ≥1.0 g/kg BW/day only lost an average of 3.7% SMI, and no additional benefit was observed beyond 1.5 g/kg BW/day protein intake. However, only a small proportion of patients (15%) had protein intake higher than 1.5 g/kg BW/day, which could be a reason that we were unable to observe the effects of higher protein intake on SMI changes.

The obesity paradox, which remains controversial within the oncological population, suggests that although obesity is implicated in development of many chronic diseases, it may conduce a survival effect in patients with some chronic diseases, including cancer [23]. Proponents of this theory would suggest that excess adiposity would convey a muscle-sparing effect in cancer patients [23]. High BMI has been linked to shorter survival in pancreatic and colorectal cancers [24,25], while also being linked to longer survival in gastrointestinal and lung cancers. Additionally, the ESPEN prognostic grading system associates higher BMIs to better prognoses [13]. In line with previous studies, higher baseline adiposity, primarily driven by subcutaneous adipose tissue mass, correlated with greater loss of muscle even after controlling for confounding variables (baseline muscularity, sex, age, stage of disease, and treatment) [15,26,27]. When evaluating subcutaneous and visceral fat independently, subcutaneous appeared to be more significantly related to muscle loss. However, in a mixed tumor group, SATI was associated with lower mortality risk [28]. The effects of adiposity on skeletal muscle mass and its underlying mechanism remain to be fully elucidated.

The strengths of this study include the longitudinal design enabling assessment over a total treatment trajectory. Assessing body composition by L3 CT imaging enabled discerning between SM, TAT, SAT, and VAT for the quantification of each depot individually [17]. However, the majority of HNC patients lack abdominal (L3) imaging with CT. This highlights the importance of developing other methods for body composition assessment in HNC patients, such as CT-measured body composition at C3 using neck CT scans [29]. This study would have benefited from information pertaining to patient response to treatment, basal metabolic rate, tumor-based metabolic abnormalities, inflammation parameters, and levels of anabolic mediators.

## 5. Conclusions

The preservation of skeletal muscle mass remains a critical therapeutic challenge in the management of the cancer patients linked to clinical and functional outcomes [30,31]. These results suggest that meeting minimum energy recommendations (25 kcal/kg BW/day) may not be sufficient to attenuate loss of SM in HNC patients. Maintaining positive energy balance is a requirement to effectively utilize protein for the protein synthesis of muscle. Further research is required to elucidate the energy and protein requirements in oncology patients, as the requirements for muscle maintenance may depend on the extent of metabolic alterations in different cancer types and treatment modalities. Cancer-associated malnutrition is a complex product of negative energy and protein balance, systemic inflammation syndrome, hypoanabolism, and tumor or inflammation derived hypercatabolism [13,32,33]. Multifactorial approaches that include physical activity have proven to have efficacy in this setting [34,35,36]. Addressing the limits in food intake is only one aspect of the approach required to prevent malnutrition and muscle wasting during treatment. The findings of this study contribute to the evidence base for recommended energy and protein intakes for cancer patients.

## Figures and Tables

**Table 1:** Patient characteristics (*n* = 41).

Characteristics	Baseline
Age, years	
Mean ± SD	57.8 ± 10.8
Range	41–84
Sex	
Male *n* (%)	32 (78)
BMI, (kg/m^2^)	
Mean ± SD	28.4 ± 5.1
Range	19.1–43.6
AJCC staging * *n* (%)	
I	1 (2)
II	3 (7)
III	21 (51)
IV	16 (39)
Tumor Site; *n* (%)	
Lip/oral cavity	15 (36)
Pharynx	22 (54)
Larynx	2 (5)
Salivary gland	2 (5)
Mode of Treatment; *n* (%)	
RT	6 (15)
Surgery RT	5 (12)
Chemoradiotherapy	25 (61)
Surgery chemoradiotherapy	5 (12)

BMI, body mass index; RT, radiotherapy. * American Joint Committee on Cancer (AJCC) Staging 7th Edition 2010 (version 01.04.00).

**Table 2:** Changes in weight, body composition, and dietary intake at baseline and post-treatment.

	Baseline	Post-Treatment	Mean ∆	*p*-Value
Weight (kg)	85.5 ± 16.4	78.5 ± 13.9	−6.9 ± 4.9	<0.001
SMI (cm^2^/m^2^)	52.2 ± 10.4	45.7 ± 8.6	−5.9 ± 4.3	<0.001
Estimated Skeletal Muscle (kg)	27.9 ± 6.1	24.8 ± 5.2	−5.3 ± 2.2	<0.001
TATI (cm^2^/m^2^)	128.0 ± 56.3	92.2 ± 49.2	−36.4 ± 38.4	<0.001
SATI (cm^2^/m^2^)	67.1 ± 41.9	51.4 ± 32.9	−18.0 ± 22.5	<0.001
VATI (cm^2^/m^2^)	57.7 ± 27.6	38.1 ± 23.5	−18.1 ± 22.8	<0.001
Energy Intake (kcal/day)	2054 ± 720	1637 ± 599	−416 ± 933	0.029
Energy Intake (kcal/kg/day)	25.1 ± 7.9	22.2 ± 9.6	−2.8 ± 11	NS
Protein Intake (g/day)	91.7 ± 34	73.9 ± 33	−17.8 ± 39.6	0.028
Protein Intake (g/kg/day)	1.1 ± 0.4	1.0 ± 0.5	−0.1 ± 0.4	NS

SMI, skeletal muscle index; SAT, subcutaneous adipose tissue; TAT, total adipose tissue; VAT, visceral adipose tissue.
